# Respective and Combined Effects of Impairments in Sensorimotor Systems and Cognition on Gait Performance: A Population-Based Cross-Sectional Study

**DOI:** 10.1371/journal.pone.0125102

**Published:** 2015-05-19

**Authors:** Olivier Beauchet, Cyrille P. Launay, Bruno Fantino, Gilles Allali, Cédric Annweiler

**Affiliations:** 1 Department of Neuroscience, Division of Geriatric Medicine, Angers University Hospital, Angers, France; 2 Department of Neurology, Geneva University Hospital and University of Geneva, Switzerland; 3 Department of Neurology, Division of Cognitive & Motor Aging, Albert Einstein College of Medicine, Yeshiva University, Bronx, New York, United States of America; 4 Robarts Research Institute, Department of Medical Biophysics, Schulich School of Medicine and Dentistry, the University of Western Ontario, London, Ontario, Canada; Banner Alzheimer's Institute, UNITED STATES

## Abstract

**Background:**

Respective and combined effects of impairments in sensorimotor systems and cognition on gait performance have not been fully studied. This study aims to describe the respective effects of impairments in muscle strength, distance vision, lower-limb proprioception and cognition on the Timed Up & Go (TUG) scores (i.e., performed TUG [pTUG], imagined TUG [iTUG] and the time difference between these two tests [delta TUG]) in older community-dwellers; and to examine their combined effects on TUG scores.

**Methods:**

Based on a cross-sectional design, 1792 community-dwellers (70.2±4.8 years; 53.6% female) were recruited. Gait performance was assessed using pTUG, iTUG and delta TUG. Participants were divided into healthy individuals and 15 subgroups of individuals according to the presence of impairment in one or more subsystems involved in gait control (i.e., muscle strength and/or distance vision and/or lower-limb proprioception and/or cognition [episodic memory and executive performance]). Impairment in muscle strength, distance vision and lower-limb proprioception was defined as being in the lowest tertile of performance. Impairment in cognition was defined as abnormal episodic memory and executive tests.

**Results:**

A total of 191 (10.7%) exhibited impairment in muscle strength, 188 (10.5%) in distance vision, 302 (16.9%) in lower-limb proprioception, and 42 (2.3%) in cognition. Linear regressions showed that cognitive impairment as well as dual combinations of impairments were associated with increased pTUG (P<0.02). Impairment in lower-limb proprioception was associated with decreased iTUG (P=0.015). All combinations of impairments, except those including muscle strength and the combinations of the 4 subsystems, were associated with increased delta TUG (P<0.04).

**Conclusion:**

Cognitive integrity is central for efficient gait control and stability, whereas lower-limb proprioception seems to be central for gait imagery.

## Introduction

Gait is the medical term use to describe human locomotion [1]. Gait is a distinctive motor attribute of an individual that changes over the life span due to the combined effect of physiological aging and morbidities, all changes leading to gait disorders [2–4]. Gait disorders cause numerous adverse outcomes such as falls and related-injuries, disability, institutionalisation and death [2–7]. Gait disorders are a worldwide major issue because of their expanding prevalence that can reach 80% in oldest-old individuals [7–9]. More information on the early stages of gait disorders is required to better understand their origin and, thus, to propose adapted preventive and curative interventions in older adults.

Gait is a dynamic balance condition in which the body's center of gravity (COG) is located above a small base of support while it moves in the horizontal plan [10]. As long the COG is maintained over the base of support, gait is stable [11]. To maintain postural stability while walking, an individual is therefore required to actively control the movements of its COG [10,11]. Several physiological sensory and motor subsystems contribute to the dynamic postural control, the most important ones identified in older adults being muscle strength, lower-limb proprioception, vision and cognition [1,4,12–14]. Age-related physiological impairment in the performance in these 4 subsystems may, therefore, modulates gait performance [1,4,12]. The impact on gait performance and gait control of the impairment of these subsystems either considered separately or in combination, has not been fully studied in older adults [12].

From the medical examination to the research settings using spatio-temporal gait analysis, several strategies of assessment have been implemented to explore gait performance and gait control [2,4,15]. Nowadays, the use of gait analysis systems is growing in clinical routine because they are user-friendly and they allow simple objective gait measurements [16,17]. In contrast, exploring the highest levels of gait control disorders is more complex in clinical practice, and limited to two alternatives: either using the dual-task paradigm [i.e., walking while simultaneously executing an attention-demanding task], or using motor imagery of gait (i.e., the mental simulation of gait without its actual execution) [2,13–16]. Recent literature has highlighted the interest of the latter approach [18–21], including the mental chronometry approach applied to a well-known motor test used in clinical routine and called the "Timed Up & Go" (TUG) test [18–22]. This standardized basic assessment of a functional mobility for basic daily living motor activity records the time needed to stand up, to walk 3 meters, to turn back and sit down [22]. It has been reported that cognitive performance, and in particular executive functioning, contributes to the temporal correspondence between executing and imaging gait in patients with neuropsychiatric conditions like dementia, schizophrenia or multiple sclerosis [18–21]. It has also been shown that older adults with cognitive impairment executed the imagined TUG test (iTUG) more quickly than they actually performed it (pTUG) [18,19]. In contrast, healthy younger adults have similar performance in both conditions [18,19]. This discrepancy in terms of performance between pTUG and iTUG, called “delta TUG”, has been interpreted as the awareness of movements and physical performance, and thus may be used as a biomaker of the disorders of the higher levels of gait control [18–21].

While the specific impact of cognitive impairment on motor imagery of gait has already been established [18–21], the respective effects of the impairments in sensorimotor subsystems (i.e., muscle strength, lower-limb proprioception and distance vision), as well as their interactions with cognitive impairment, have not been studied yet. We hypothesized that impairments in sensorimotor systems and cognition could affect gait performance and the motor imagery of gait. This study aims 1) to describe the respective effects of impairments in muscle strength, distance vision, lower-limb proprioception and cognition on the TUG scores (i.e., pTUG, iTUG, and delta TUG) in older community-dwellers without dementia; and 2) to examine their combined effects on TUG scores.

## Methods

### Population selection and study design

Between January 2008 and April 2012, 4192 older community-dwellers were recruited in 8 French Health Examination Centers (HEC) in Eastern France during a free and full medical examination. Sampling and data collection procedures have been described elsewhere in detail [23]. The study was based on a cross-sectional design. The exclusion criteria were: inability to understand and speak French, an acute medical illness in the past 3 months; neurological diseases such as Parkinson’s disease, cerebellar disease, myelopathy, peripheral neuropathy; major orthopedic diagnoses (e.g., osteoarthritis) involving the lumbar vertebra, pelvis or lower extremities, inability to walk 6 meters unassisted and being younger than 65 years of age. The exclusion criteria for the present analysis were: missing values, dementia, depression symptoms defined as a score of the 4-item geriatric depression scale (GDS) ≥1, institutionalization and the use of walking aids [24].

From the initial 4192 individuals, 1792 (42.8%) were included in the present analysis. Participants were separated into 16 subgroups based on their performance on handgrip strength, distance vision, lower-limb proprioception and cognition. A total of 384 (21.4%) participants with no impairments defined the group of healthy individuals (HI), which was subsequently used as the reference group. The remaining 1408 participants (78.6%) were divided into 15 subgroups according to the combinations of impairments in the 4 subsystems (i.e., muscle strength and/or distance vision and/or lower-limb proprioception and/or cognition). Impairments in muscle strength, distance vision, lower-limb proprioception were defined as being in the lowest tertile of performance. The other two tertiles combined were used to define the normal performance. A short mini-mental state examination (S-MMSE) score ≤5 combined with one or more errors made in the execution of the clock-drawing test were used to define cognitive impairment [25,26].

### Clinical and Gait Assessment

Clinical assessment corresponded to a full medical examination along with collecting age, gender, and measures of height and weight. The number of drug classes taken daily was also recorded. Body mass index (BMI, in kg/m^2^) was calculated based on anthropometry measurements (i.e., weight in kilograms and height in meters). The maximal isometric voluntary contraction (MVC) strength of hand was measured with computerized hydraulic dynamometers (Martin Vigorimeter, Medizin Tecnik, Tutlingen, Germany). The test was performed three times with the dominant arm. The mean value of MVC of all trials was used in the present data analysis. Distance binocular vision was measured at 5 m with a standard Monoyer letter chart and score from 0 (i.e., worst performance) to 10 (i.e., best performance) [27]. Vision was assessed with corrective lenses on if needed. Lower-limb proprioception was evaluated with a graduated tuning fork placed on the tibial tuberosity measuring vibration threshold [28]. The participants were asked to indicate when the vibration stimulus was felt for the first time (perception threshold) and when this stimulus disappeared (disappearance threshold). The latter value was used in our study and ranged between 0 (i.e., worst performance) to 8 (i.e., best performance). The mean value obtained for the left and right sides was used in the present data analysis. Cognition was evaluated with two cognitive tests. First, the S-MMSE was used to explore the episodic memory [25]. Its score ranged from 0 (i.e., worst performance) to 6 (i.e., best performance) and a score ≤5 was considered as impairment in episodic memory performance [25]. Second, the clock-drawing test (CDT) was used to examine executive function [26]. A low executive performance was considered if one or more errors were made in the execution of drawing the face of the clock and/or the hands of the clock.

Regarding the gait assessment, individuals were asked to perform the TUG at their self-selected normal speed in a well-lit environment. They all completed one trial for the TUG and then followed by the imagery of TUG while sitting in a chair. The times for each condition were recorded with a stopwatch to the nearest 0.01second. Before testing, a trained evaluator gave standardized verbal instructions regarding the test procedure. Individuals were seated, allowed to use the armrests to stand up and instructed to walk three meters, turn around, walk back to the chair and sit down. The stopwatch was started on the command “ready-set-go” and stopped as the participant sat down. For the imagined condition, individuals sat in the chair and were instructed to imagine performing the TUG (iTUG) and to say “stop” out loud when they were finished. Individuals could choose to do the iTUG with their eyes opened or closed, and they were not instructed on the choice of the modality of mental imagery.

### Standard Protocol Approvals, Registrations, and Patient Consents

Individuals were included after having given their written informed consent for research. The study was conducted in accordance with the ethical standards set forth in the Helsinki Declaration (1983). Lyon Sud-Est III local Ethical Committee, France, approved the study protocol.

### Statistical analysis

The participants’ characteristics were summarized using means and standard deviations or frequencies and percentages, as appropriate. Normality of data distribution was checked using a skewness-kurtosis test. As the number of observations was > 40 for each subgroup, no transformations were applied to the variables of interest. For the current analysis, delta TUG was calculated from the following formula: (Timed up & Go realized—Timed up & Go imagined / ((Timed up & Go realized—Timed up & Go imagined) /2) x100.

First, comparisons between HI, used as the reference group, and subgroups with impairments were performed using unpaired *t*-test or Chi-square test, as appropriate. Due to Bonferroni’s correction applied to adjust for multiple comparisons, P-values <0.004 were considered as statistically significant for between-group comparisons. Second, multiple linear regression analyses were performed to examine the association between TUG scores (i.e., pTUG, iTUG and delta TUG) used as the dependent variables and the previous 15 subgroups of individuals with impairments in subsystems (i.e., muscle strength, distance vision, lower limb proprioception and cognition) used as the independent variables adjusted on participants’ clinical characteristics (i.e., age, gender, number of drug classes daily taken and BMI). P-values less than 0.05 were considered as statistically significant for the linear regression analyses. All statistics were performed using SPSS (version 19.0; SPSS, Inc., Chicago, IL).

## Results

Table 1 shows the clinical characteristics of participants according to their impairment in each subsystem. The prevalence of impairment in performance of subsystems was as followed: individuals with muscle strength impairment (IMI) (n = 191, 10.7%), individuals with distance vision impairment (IVI) (n = 188, 10.5%), individuals with lower limb proprioception impairment (IPI) (n = 302, 16.9%) and individuals with cognitive impairment (ICI) (n = 42, 2.3%). Impairment in performance in a subsystem when pooling all participants with impairment, whatever its nature, was associated with increased age, gender male, a higher number of drug classes daily taken and higher pTUG and delta TUG scores compared to the HI group (all P-values <0.001). IMI were older (P<0.001), more frequently women (P<0.001) and took more drug classes (P<0.001). They had also a worse distance vision score (P = 0.001) and lower-limb proprioception score (P = 0.003), and a higher pTUG score (P<0.001) compared to HI. IVI were also older (P<0.001), had more frequently an abnormal CDT score (P<0.001) and trended to have greater pTUG score (P = 0.005) compared to HI. IPI were older (P<0.001) and more frequently women (P<0.001). They had also greater strength (P = 0.003) and delta TUG score (P<0.001) compared to HI. ICI trended to have greater pTUG (P = 0.024) and iTUG (P = 0.027) scores compared to HI.

**Table 1:** Participants' characteristics separated by impairment in the four subsystems (i.e., muscle strength, distance vision, lower-limb proprioception and cognition) involved in gait control (n = 1792).

						Participants with impairment					
	Healthy participantsused as the reference group (n = 384)	in one or more subsystems * (n = 1408)	P-value†	In handgrip strength‡ (n = 191)	P-value†	In distance vision‡ (n = 188)	P-value†	In lower-limb proprioception‡ (n = 302)	P-value†	In cognition§ (n = 42)	P-value†
Age (years), mean±SD	68.3±3.3	70.7±5.0	**<0.001**	69.5±3.9	**<0.001**	70.1±4.8	**<0.001**	69.5±4.2	**<0.001**	68.3±3.6	0.981
Women, n (%)	270 (70.3)	690 (49.0)	**<0.001**	191 (96.9)	**<0.001**	130 (69.1)	0.776	257 (85.1)	**<0.001**	30 (71.4)	0.880
Number of therapeutic classes per day, mean±SD	2.2±2.1	2.9±2.4	**<0.001**	3.0±2.3	**<0.001**	2.5±2.4	0.047	2.5±2.3	0.027	2.8±2.4	0.071
BMI (kg/m2), mean±SD	26.4±3.9	26.4±4.3	0.752	25.6±4.8	0.020	26.0±3.6	0.173	27.1±4.1	0.041	26.7±3.8	0.693
Handgrip strength# (N.m^-2^), mean ± SD	37.5±8.5	30.1±10.7	**-**	20.3±3.2	**-**	35.6±7.9	0.010	39.4±8.3	**0.003**	35.8±8.4	0.216
Distance vision score¶ (/10), mean ± SD	8.7±1.0	6.8±2.2	**-**	8.4±1.0	**0.001**	5.1±1.2	**-**	8.7±1.0	0.573	8.7±1.0	0.896
Lower-limb proprioception score|| (/8), mean ± SD	7.4±0.5	6.0±1.7	**-**	7.5±0.5	**0.003**	7.4±0.6	0.562	5.0±1.6	**-**	7.2±0.6	0.091
S-MMSE score (/6), mean±SD	5.7±0.7	5.6±0.7	**-**	5.7±0.7	0.775	5.5±0.9	0.006	5.7±0.7	0.307	5.7±0.7	-
Abnormal clock drawing test **, n (%)	10 (2.6)	305 (21.7)	**-**	11 (5.8)	0.057	18 (9.6)	**<0.001**	21 (7.0)	0.006	42 (100)	-
Timed Up & Go, mean ±SD											
pTUG, (s)	9.0±1.8	9.9±2.3	**<0.001**	9.7±2.0	**<0.001**	9.5±2.0	0.005	9.3±2.1	0.059	9.7±2.3	0.024
iTUG (s)	6.8±2.3	6.8±2.9	0.896	7.1±2.8	0.209	7.0±3.4	0.470	6.5±3.0	0.123	6.7±3.1	0.794
Delta TUG†† (%)	31.2±27.9	40.6±33.7	**<0.001**	34.9±33.9	0.163	36.6±36.3	0.052	40.6±33.1	**<0.001**	41.6±34.9	0.027

SD: Standard deviation

BMI: Body mass index

TUG: Timed Up & Go

pTUG: Performed Timed Up & Go

iTUG: Imagined Timed Up & Go

*: Group composed of the 723 participants with impairment in one subsystem and of the 685 participants with impairments in more than one subsystem

^†^: Comparison with healthy participants based on unpaired t-test or Chi square test, as appropriate

^‡^: Impairment in muscle strength, distance vision, lower-limb proprioception was defined as being in the lowest tertile of performance. The other two tertiles combined were used to define normal performance.

^§^: Combination of episodic memory impairment (i.e., short mini-mental status examination score ≤5/6) and executive impairment (i.e., one or more errors made in the execution of drawing the face of the clock and/or the hands of the clock).

^#^: Mean value of 3 trials measuring the maximal isometric voluntary contraction strength measured with computerized dynamometers expressed in Newton per square meter

^¶^: Binocular vision acuity at distance of 5 m with a Snellen letter test chart

^||^: Mean value of left and right side and based on graduated tuning fork placed on the tibial tuberosity measuring vibration threshold

**: Considered if one or more errors were made in the execution of drawing the face of the clock and/or the hands of the clock

^††^: Calculated from the following formula: [(Timed “Up & Go—Timed “Up & Go” imagined) / (Timed “Up & Go” + Timed “Up & Go” imagined) / 2] x 100

P-value significant with Bonferroni’s correction (i.e., P<0.004) indicated in bold.

**Fig 1:**
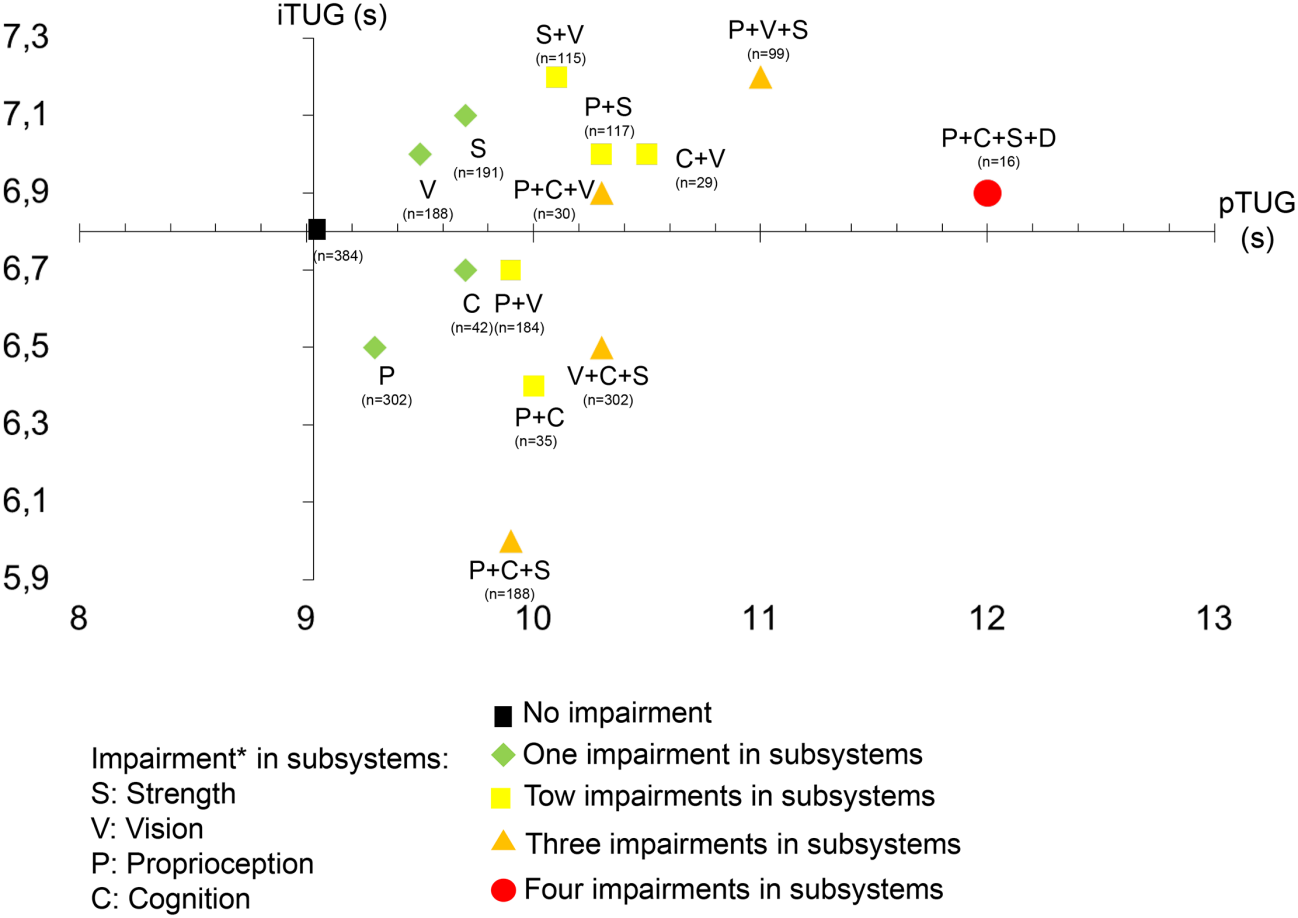
Representation of interdependence between performed Timed Up & Go test on x-axis and imagined Timed Up & Go on the y-axis among the different subgroups of participants combining or not impairment in one or more subsystem (i.e., muscle strength, distance vision, lower limb proprioception and cognition). *: Impairment in muscle strength, distance vision, lower-limb proprioception was defined as being in the lowest tertile of performance. The other two tertiles combined were used to define normal performance. Combination of episodic memory impairment (i.e., short mini-mental state examination score ≤5/6) and executive impairment (i.e., one or more errors made in the execution of drawing the face of the clock and/or the hands of the clock) was used to define cognitive impairment. TUG: Timed Up & Go. pTUG: Performed Timed Up & Go. iTUG: Imagined Timed Up & Go. s: Second.

Fig 1 is a graphical representation of interdependence of pTUG on the x-axis and of iTUG on the y-axis. It shows that largest discrepancies between pTUG and iTUG (i.e. worse performance) were found with impairment in lower-limb proprioception following by cognitive impairment, association of impairments in proprioception and in vision or cognition, and combination of impairments in proprioception, cognition and muscle strength. In final, combination of impairments in vision, cognition and muscle strength corresponded to worse performance.

**Fig 2:**
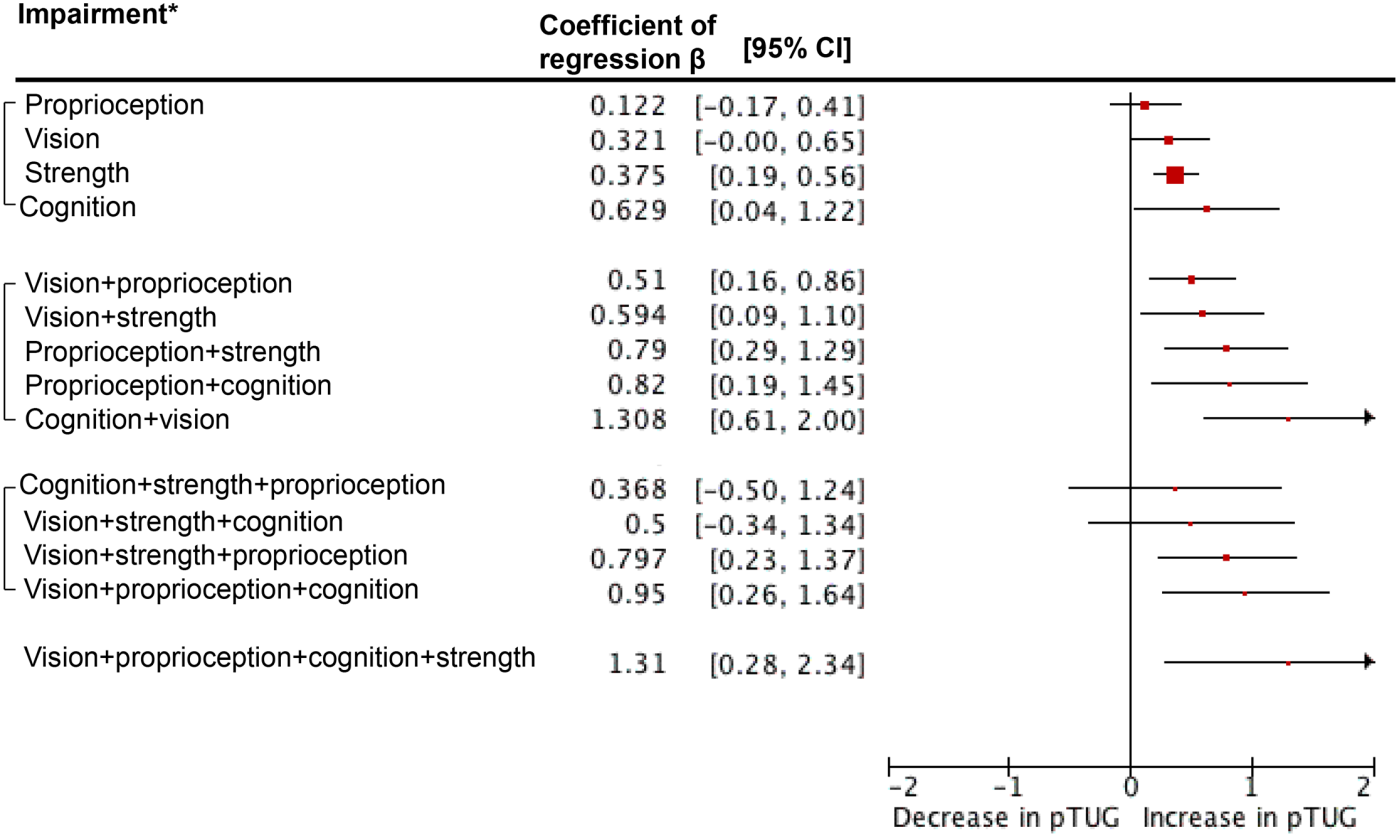
Multiple linear regression analyses showing associations between performed timed up and go test used as dependent variables and subgroups of individuals combining or not decline in performance in different subsystems (i.e., muscle strength, distance vision, lower-limb proprioception and cognition) used as independent variables adjusted on individuals’ clinical characteristics. TUG: Timed Up & Go. pTUG: Performed Timed Up & Go. Horizontal lines are the 95% confidence intervals extending positive and negative from the beta value. Healthy individuals are used as the reference group and correspond to the vertical axis. *: Impairment in muscle strength, distance vision, lower-limb proprioception was defined as being in the lowest tertile of performance. The other two tertiles combined were used to define normal performance. Combination of episodic memory impairment (i.e., short mini-mental state examination score ≤5/6) and executive impairment (i.e., one or more errors made in the execution of drawing the face of the clock and/or the hands of the clock) was used to define cognitive impairment. All multiple linear regression analyses were adjusted on individuals’ clinical characteristics (i.e., age, gender, number of drug classes daily taken and body mass index).

**Fig 3:**
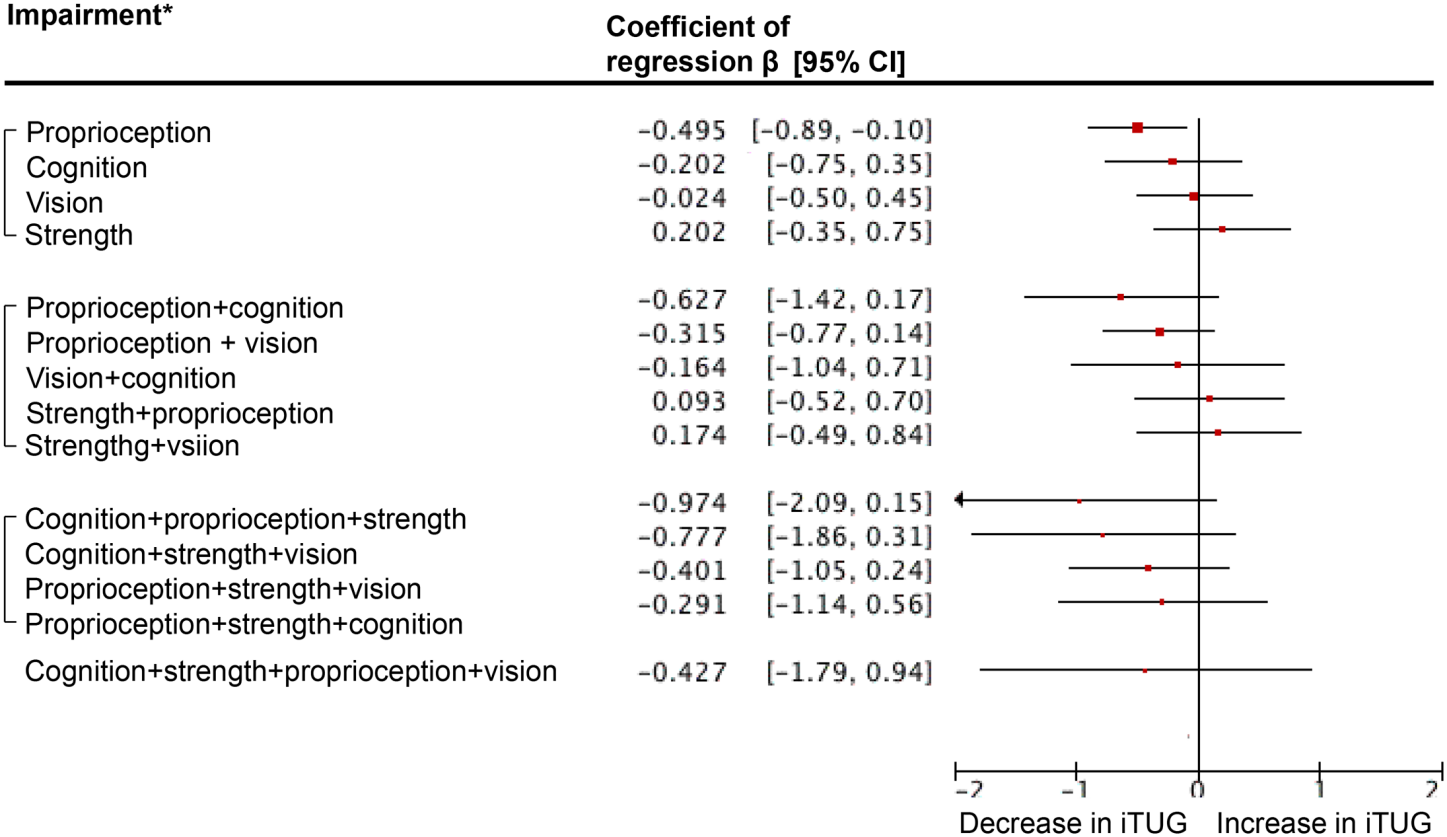
Multiple linear regression analyses showing associations between imagined timed up and go test used as dependent variables and subgroups of individuals combining or not decline in performance in different subsystems (i.e., muscle strength, distance vision, lower-limb proprioception and cognition) used as independent variables adjusted on individuals’ clinical characteristics. TUG: Timed Up & Go. iTUG: Imagined Timed Up & Go. Healthy individuals are used as the reference group and correspond to the vertical axis. *: Impairment in muscle strength, distance vision, lower-limb proprioception was defined as being in the lowest tertile of performance. The other two tertiles combined were used to define normal performance. Combination of episodic memory impairment (i.e., short mini-mental state examination score ≤5/6) and executive impairment (i.e., one or more errors made in the execution of drawing the face of the clock and/or the hands of the clock) was used to define cognitive impairment. All multiple linear regression analyses were adjusted on individuals’ clinical characteristics (i.e., age, gender, number of drug classes daily taken and body mass index).

**Fig 4:**
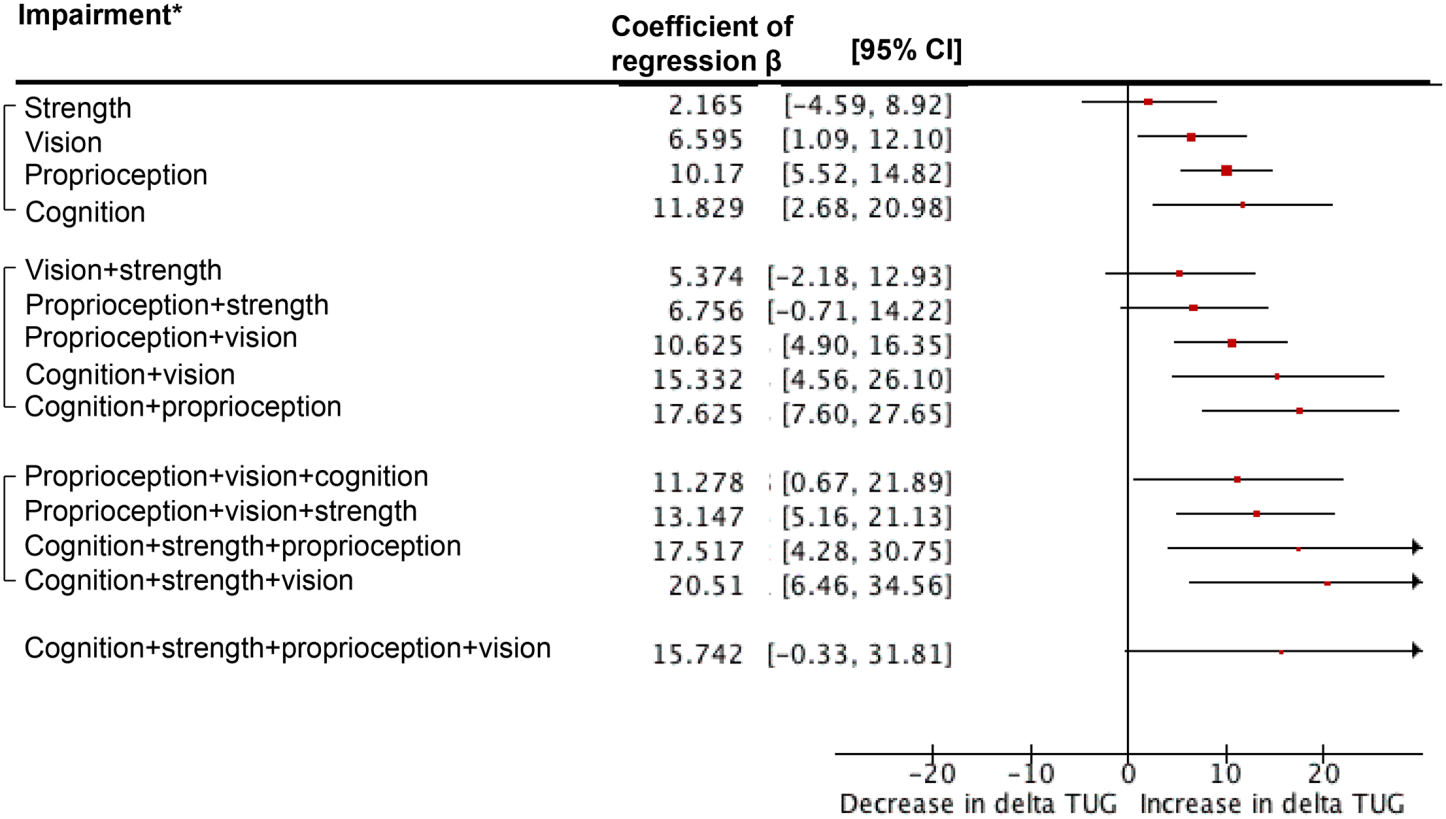
Multiple linear regression analyses showing associations between delta timed up and go test used as dependent variables and subgroups of individuals combining or not decline in performance in different subsystems (i.e., muscle strength, distance vision, lower-limb proprioception and cognition) used as independent variables adjusted on individuals’ clinical characteristics. TUG: Timed Up & Go. Delta TUG: Calculated from the following formula: [(performed Timed “Up & Go—Timed “Up & Go” imagined) / (performed Timed “Up & Go” + Timed “Up & Go” imagined) / 2] x 100. Horizontal lines are the 95% confidence intervals extending positive and negative from the beta value. Healthy individuals are used as the reference group and correspond to the vertical axis. *: Impairment in muscle strength, distance vision, lower-limb proprioception was defined as being in the lowest tertile of performance. The other two tertiles combined were used to define normal performance. Combination of episodic memory impairment (i.e., short mini-mental state examination score ≤5/6) and executive impairment (i.e., one or more errors made in the execution of drawing the face of the clock and/or the hands of the clock) was used to define cognitive impairment. All multiple linear regression analyses were adjusted on individuals’ clinical characteristics (i.e., age, gender, number of drug classes daily taken and body mass index).

Figs 2, 3 and 4 show the results of multiple linear regressions exploring associations between impairment in subsystems and scores of pTUG, iTUG and delta TUG. For pTUG and when considering impairment in one subsystem only, cognitive impairment (P = 0.036) and muscle strength impairment (P = 0.021) were associated with increase in pTUG time. In contrast, all dual combinations of impairments were associated with increase in pTUG time (P<0.03). In addition, combinations of impairments in distance vision plus muscle strength plus lower-proprioception (P = 0.006) and distance vision plus lower-proprioception plus cognition (P = 0.007) were associated with increase in pTUG time. For iTUG, only impairment in lower-limb proprioception was associated with a decrease in iTUG (P = 0.015). For delta TUG, strength impairment alone or combined with another impairment in one subsystem was not significant (P>0.07). All other combinations were significantly associated with increase in delta TUG (P<0.04), except the one combining impairments in the 4 subsystems (P = 0.055).

## Discussion

Our findings show that cognitive impairment considered either separately or in combination with any other subsystem impairment, notably muscle strength, was strongly associated with greater (i.e. worse performance) pTUG and delta TUG scores. In contrast, lower-limb proprioception impairment was associated with lower (i.e. worse performance) iTUG score. We also found that impairments in all subsystems were associated with worse delta TUG score with a tendency to an increased effect when there was an accumulation of impairments, the highest impact being shown when combining cognition and muscle strength.

Our study confirms that the association between cognition and gait control in older individuals without dementia. The past decade has brought accumulating evidence that impairment in cognitive performance in demented and non-demented individuals resulted in deterioration of gait performance [2,13–15]. Commonly described in later stages of dementia, worsening of gait performance has also been reported early in the progression of dementia and even at the prodromal stage of mild cognitive impairment (MCI) [29–34]. Furthermore, it has been shown that both memory and executive function (EF) are particularly involved on gait control, even in routine walking condition [33–37]. Nowadays, evidence of an association of EF and memory with gait control comes from clinical and brain imaging studies. First, although few studies have explored the association of EF with gait performance among healthy older adults, these studies showed similar results [33–36]. It has been reported that low global EF performance was associated with a low gait performance, and more precisely with low gait speed and high stride-to-stride variability [33,35,36]. Furthermore, an association between increased gait variability and decreased performances in information updating and monitoring, which is a specific subdomain of EF, has been shown in healthy older adults [34]. It was suggested that this involvement of EF could be explained by age-related changes in sensorimotor system leading to a decrement in automaticity compensated by an increased involvement of EF to properly process all sensorimotor information. In addition to these clinical data, studies reported that abnormalities (e.g., morphological and functional) of frontal lobe was related to lower gait performance in HI [38–40]. Second, there is increased evidence that memory is also strongly involved in gait control in older adults. Indeed, higher episodic memory performance has been associated to higher gait speed while single and dual tasking in older adults [41]. Lower hippocampal volume has been associated with greater gait variability [39]. Recently Shimada et al. reported that greater desactivation in the white matter of hippocampus was related to greater gait variability in healthy older adults using positron emission tomography brain imaging, this result being in concordance with previous results showing that increased gait variability was associated with poorer hippocampal metabolism in a similar population [40,42]. The strong association between EF, memory and gait control in HI could be explained by the fact that the frontal lobe is a brain structure involved in simulating motor actions and that the hippocampus has an important role in the timing for the rhythmicity of locomotion and gait navigation.

Our results underscored that lower-limb proprioception was strongly related to iTUG performance. Indeed, impairment in lower-limb proprioception was associated with a decreased iTUG time. iTUG is a motor imagery test that simulates mentally an action without its actual execution. This approach was widely used to study higher-level control of action, based on mental chronometry, which is a tool in neuroscience to measure the time course of mental operations [43,44]. A close temporal correspondence between executing and imaging gait has been previously reported in healthy young subjects [45,46]. Furthermore, this close correspondence in timing the mental simulation of gait and its actual execution reflects the functioning of a cerebral network involving the primary motor cortex as well the prefrontal area BA 10 that participates in higher-order gait control [47]. Therefore, a lower time while imaging TUG compared to executing it is considered as a biomarker of disorders of the higher levels of gait control [19–21]. This association between lower proprioception and decreased iTUG highlighted a strong association between simulating an action and the body perception in the space. Proprioception refers to the sense of knowing where one's body is located in space [48]. In our study we accessed lower-limb proprioception by using a graduated tuning fork placed on the tibial tuberosity, which is the best way to measure the ability to sense the static position of a joint or limb segment [48]. There is a wealth literature on the importance of proprioception feedback in the control of movements: impairment in proprioception provoking degradation in movement performance and in particular in postural movement control [49]. Impairment in proprioception function may influence cognitive performance. For instance, it has been shown that when the quality of proprioceptive feedback was reduced in older HI under a dual-task condition (i.e., maintaining of static position while performing an attention-demanding task) they sacrificed performance on the cognitive task in favour of maintaining postural stability [50,51]. The age-related central processing deficit related to proprioception feedback reported in clinical studies has been confirmed by functional brain imaging studies that showed neural correlates. For instance, hand-foot interlimb coordination has been associated with an extended network of neural activation which included secondary somatosensory area as well as sensory integration areas located in superior temporal and supramarginal gyri [52]. More recently, we found an association between higher STV and lower gray matter volume in the right parietal lobe in older HI [53]. In addition, it is now established that the parietal lobe is able to map objects perceived visually into body coordinate positions [54]. Taken together, this strong association between proprioception and gait control could explain that lower-limb proprioception impairment resulted in altered mental imagery of gait in the present study.

Our results also showed a complex interplay between impairments in locomotion subsystems. We observed a deterioration of motor imagery of gait assessed by delta TUG, when the impairment of subsystems increases, except when all impairments in equilibration systems were combined. Furthermore, worse gait performance was reported when cognition was respectively associated with impairment in strength for pTUG and impairment in lower-limb proprioception for iTUG. Interestingly, the combination of cognitive and strength impairments provoked an important deterioration of gait performance exceeding a single additional effect. Such effect could be explained by the fact that cognition and muscle strength are the most important equilibration systems involved in gait execution. It is well established that performance in realized movement strongly depends on the integrity of muscles [50–52]. In contrast, the relation with cognition remains unclear. It could be suggested that the best performance in realized movement depends not only on the high peripheral ability to perform it, but also on the cognitive ability to control it. In addition, a similar reasoning may be proposed to explain the specific effect of the combination of cognitive and proprioception impairments on the internal simulation of gait. Indeed, proprioception and cognition are both key components of gait imagery.

Some limitations of our study need to be acknowledged. First, the cross-sectional design may be problematic when exploring an association between gait and impairments in locomotors subsystems compared to a prospective cohort study design. Indeed, the causality and direction of the associations should be carefully interpreted. Second, abnormal scores on the S-MMSE and the clock drawing test could be not sufficient to diagnose satisfactorily memory and executive impairments. These tests are usually used as screening tests rather than diagnostic tools in general population. A diagnosis of cognitive impairment in these two sub-domains usually requires a multidisciplinary meeting involving geriatricians, neurologists and neuropsychologists during which the results of neuropsychological assessment, medical examination, blood tests and brain imaging are discussed. Third, although we were able to control for many characteristics likely to modify the association between gait and cognitive performance, residual potential confounders might still be present in our study. In contrast, our study has a number of strengths. First, to our knowledge, this is the largest population-based study in older adults to examine the association of gait performance with equilibration systems impairments. Second, compared to previous published studies, the major potential confounders (i.e., age, gender, and BMI) were taken into account in our study. Third, all participants had a comprehensive clinical examination that allowed fine categorization of individuals into the different subgroups of impairments.

## Conclusions

Cognitive impairment was associated with worse gait performance and motor imagery of gait, which suggests that cognitive integrity is central for efficient gait control and stability. Furthermore, lower-limb proprioception seems to be central for gait imagery. Combination of cognitive impairment with lower-limb proprioception and with strength affected imagination and execution of gait, respectively. These findings could be useful for the development of new preventive and curative interventions dedicated to the improvement of gait stability in older adults.
